# Rust Conversion of Proanthocyanidins to Archaeological Steel: A Case Study of Lingzhao Xuan in the Forbidden City

**DOI:** 10.3390/molecules27227711

**Published:** 2022-11-09

**Authors:** Minghao Jia, Pei Hu, Xiaogu Zhang, Gang Hu

**Affiliations:** 1 School of Archaeology and Museology, Peking University, Beijing 100871, China; 2 The Palace Museum, Beijing 100009, China

**Keywords:** proanthocyanidins, archaeological steel, rust conversion, heritage conservation

## Abstract

This work was focused on the rust conversion of proanthocyanidins (PC) for goethite (α-FeOOH), akaganeite (β-FeOOH) and lepidocrocite (γ-FeOOH), trying to show the potential of PC as an eco-friendly corrosion inhibitor and rust converter for archaeological steel conservation. The experiment used a rusted steel screw from Lingzhao Xuan of the Forbidden City in the Qing Dynasty and three kinds of pure iron oxyhydroxides as research samples. By means of micro-Raman, FTIR, XRD, XPS, SEM and EIS, PC had the ability to chemically react with iron oxyhydroxides in the rust, forming amorphous PC-FeOOH with a marked signal about 1384 cm^−1^ as phenolic-Fe in infrared properties. The original relatively stable iron oxides were not induced to phase transformation and still remained. The converted rust layer could be more stable in the corrosive medium and increased the corrosion potential more effectively. Both the rust layer resistance and the charge transfer resistance of the archaeological samples were improved by at least 3 times with 5.0 g/L of PC, which could reasonably stabilize the archaeological rust and hindered external corrosive penetration into the core. It was a mild protection material that showed satisfactory performance for archaeological steel cultural heritage and has a good application prospect.

## 1. Introduction

Archaeological metal is an important member in the field of historical exploration, and in particular, ancient large-scale metal buildings or monuments represent the essence of ancient industrial development. Therefore, cross-studies in the field of archaeology and corrosion have also been derived to analyze the deterioration mechanism of ancient metal materials and choose protection reagents. Various research and protection of outdoor metal heritage is a notable spot in the study of cultural heritage today [1,2]. Archaeological iron or steel is generally more brittle and corroded than other metal objects due to metal activity and the natural environment of alternating wet and dry [3,4]. In order to slow down the continuous corrosion and deterioration of rusted steel, it is necessary to select corrosion inhibitors and rust converters on the basis of traditional protection processes, which is one of the reasonable means that can be achieved at present [5,6].

The concept of conservation is to preserve as much authenticity and originality as possible. It is true that the protection of metal cultural heritage is not like an operation such as industrial cleaning. It is not recommended to completely remove the surface rust, but it is more inclined to preserve the metal core and rust together, because the rust layer is still a trace that can reflect historical changes [7]. Therefore, based on inhibiting the corrosion of the internal core, metal objects need more to ensure that the rust layer is stabilized by mild reagents. That is, the more active rust should be transformed into harmless components that do not react with corrosive factors in the environment, while retaining the inherently inert rust products in the original rust layer [8].

As the largest royal building complex in China, the Forbidden City has left a large amount of outdoor metal cultural heritage. Among this group, Lingzhao Xuan is located in the Yanxi Palace of the Forbidden City in Beijing and is also known as “Wondrous Pond Pavilion” [9]. It has a typical Western-style architecture that is rare in ancient China. It is one of the earliest steel/masonry composite structures built in China and has important historical, artistic and scientific value. The metal structure adapts the construction technology of cast-iron columns and hot-rolled steel beams, which were advanced in the world at that time. However, due to long-term exposure to natural wind and rain, the building has many potential safety hazards that need to be settled urgently.

The metal parts of Lingzhao Xuan can be divided into the following three categories according to their different materials and production methods: casting components represented by columns and fence decorations; steel I-beams for load-bearing; machined screws, bolts and nuts for main connection [10]. They are mainly subjected to electrochemical corrosion caused by alternating dry and wet in the atmospheric environment. Their own materials and processing characteristics lead to different degrees of corrosion, which increases the complexity and operational difficulty of cultural heritage protection.

As for the inference of corrosion mechanism, metals have different forms of mechanism conclusions due to their various compositions. Wide variation of the chemical composition of the Cu-based alloys can be distinguished as low and high tin, and can also be called leaded bronzes, copper and copper–iron alloys. Several articles have shown that the corrosion and degradation of the bronze archaeological artefacts can be attributed to their intrinsic metallurgical features and thermal treatments [11,12]. These causes induce crystallization and segregation phenomena of the impurities along the grain boundaries and could cause mechanical weakness and increase the internal corrosion phenomena [13], while for steel or iron artefacts, corrosion mechanisms still depend on every local microstructure. It can be driven locally by galvanic coupling between ferrite and cementite [14]. Many articles have focused on the structure and composition of the iron or steel rust layer [15,16], which will help scholars analyze the change of soil and atmospheric properties from an archaeological point of view. However, for cultural heritage conservationists, the target objects and necessity of rust conversion will be clearer [17]. In the atmosphere, the long-term dry and wet alternating conditions will easily cause the steel to form thick rust with various phases: oxides, oxyhydroxides, sulfides, sulfates, chlorides, carbonates, etc. In addition, the dominant phases of rust are usually associated with goethite (α-FeOOH), akaganeite (β-FeOOH), lepidocrocite (γ-FeOOH), maghemite (γ-Fe_2_O_3_) and magnetite (Fe_3_O_4_) [18,19,20]. The transformation of different phases will cause the shrinkage or expansion of the crystal sizes and subsequently induce the local internal stress to cause the peeling and cracking of the rust layer [21]. Meanwhile, the rust layer is usually not protective because the galvanic coupling effect between the steel matrix and the rust can accelerate metal corrosion, which will damage the original state of the artefacts [22]. The rust composition and distribution of archaeological steels are always more complicated than laboratory simulations, especially when exposed to natural atmospheric corrosion for more than 200 years. Therefore, the stabilized conversion and corrosion inhibition treatment of the rust layer are imperative.

The field of cultural heritage protection prefers to use green-plant reagents because they have the advantages of being biodegradable, inexpensive and non-toxic while playing the role of corrosion inhibition [23]. These advantages have inspired numerous studies on the application of plant extracts for metal corrosion [24,25]. Proanthocyanidins (PC) are a common kind of the seed extract mentioned in most literature [26], which is also known as condensed tannin. PC include a large series of oligomers and polymers of the monomeric flavan-3-ols, which vary in the attachment sites and number of oligomers [27]. This is a type of polyphenolic compound widely distributed in fruits and vegetables, especially in grape seeds [28]. The pharmaceutical industries fully utilize its excellent biological properties for the actual production [29].

In the literature, PC researchers have attempted to explore the corrosion inhibition of mild steel in hydrochloric acid solutions, and their synergistic effects with surfactants have been evaluated [30,31,32]. Generally, natural reagents are abundant organic compounds obtained from our life that have primarily flavonoids, glycosides, phenolic acids, alkaloids, etc. Most of these molecules have centers for π-electrons and electro-negative functional groups (such as –C=C–, –OH, –COOH, and –NH_2_) in triple or conjugated double bonds [33]. These structures can donate electrons to vacant d-orbitals of iron atoms to form covalent bonds and promote the physical or chemical adsorption of organic molecules at an iron interface [34]. By interacting with a metal surface to form an inert film, they can lead to a better inhibition of anodic iron oxidation and cathodic oxygen reduction for a lower metal corrosion rate [35]. PC contain electronegative oxygen atoms, hydroxyl groups and π-bonds conjugation that may provide the necessary active centers for the interaction with the steel surface, and thus this has full potential for corrosion inhibition. The anti-corrosion behavior of green inhibitors is also carried out by the rust conversion, which is able to effectively convert the iron rust into a more stable layer, serving as a barrier to block corrosive media [36]. The corrosion inhibition of archaeological steel is completely different from the smooth naked steel in the industrial field, and it is more about the conversion of iron oxyhydroxides or other active phases. Therefore, our focus should be to convert the corroded surface leading to its passivation and eliminate possible further attack on the interior metal by external corrosive media. Considering that the PC inhibitors still have a long way to go before they are put into use in industry, we can positively test this in the practice of conservation study. The application to metal cultural heritage is a potential field, which fits well with the behavior of mild and eco-friendly PC treatment.

**Figure 1:**
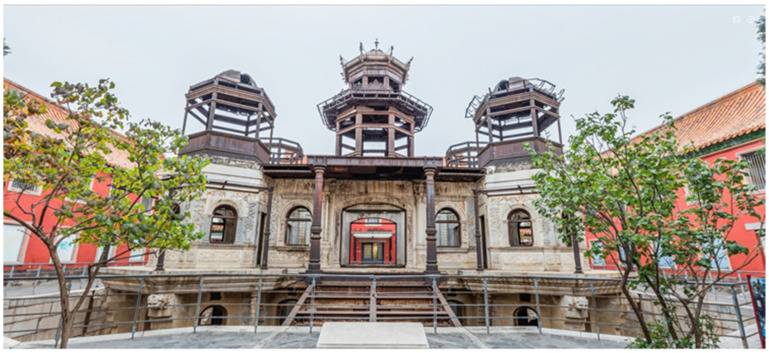
The photo of Lingzhao Xuan of the Forbidden City.

Therefore, this article has focused on the rust conversion behavior of proanthocyanidins for goethite (α-FeOOH), akaganeite (β-FeOOH) and lepidocrocite (γ-FeOOH), trying to inhibit further corrosion of archaeological steel. The experiment used steel objects corroded naturally in Lingzhao Xuan of the Forbidden City in the Qing Dynasty as research samples. The metallographic structure of the material was judged with an optical stereomicroscope. The composition and layer structure of the original rust were analyzed by SEM and Raman spectroscopy. The mechanism of PC on rust conversion was interpreted by XRD, FTIR and XPS. The improved corrosion inhibition of the modified rust layer was detected by EIS. We believed that taking three kinds of pure iron oxyhydroxides and real archaeological steel as samples could more directly prove the oxyhydroxides conversion with proanthocyanidins and more truly reflect the protection potential of proanthocyanidins for steel cultural heritage. Figure 1 is a photo of Lingzhao Xuan of the Forbidden City.

## 2. Results and Discussion

### 2.1. Metallographic Structure

**Figure 2:**
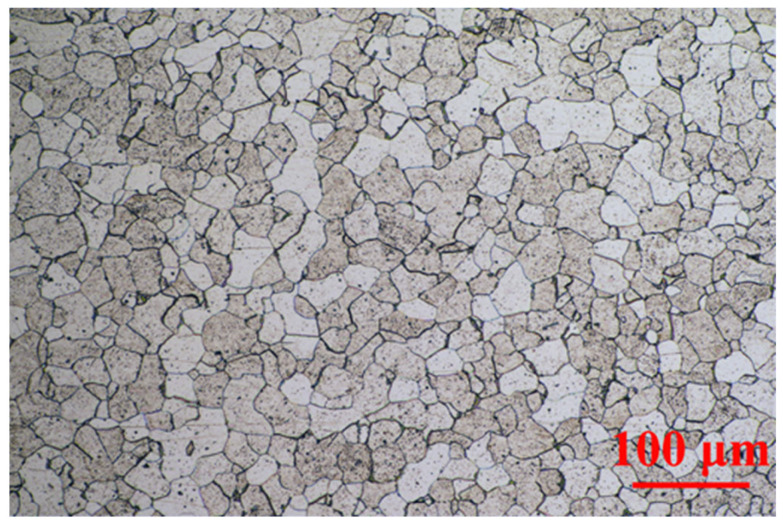
Metallographic diagrams of archaeological steel screw.

From the metallographic structure in Figure 2, the archaeological screw was ferrite-based low-carbon steel. The light-colored part was ferrite, the dark-colored part was pearlite, and the edge of the grid was cementite. The size of ferrite varied greatly locally, but the overall distribution was uniform. There were many particle inclusions inside. The elemental analysis was tested by EDS, and the mass ratio of main elements was 0.24% C, 0.06% Si, 0.15% P, 0.16% S and 92.95% Fe. Generally, structural steel with carbon content between 0.0218–0.77% could be called hypoeutectoid steel. Within this range, the lower carbon content could be regarded the lower proportion of pearlite in the steel structure [37]. Therefore, the plasticity was better, but the strength and hardness of the steel were lower. Coupled with the shear force and tensile stress for hundreds of years, it was easy for it to fall off and fracture under the action of corrosion.

### 2.2. Original Rust Composition

**Figure 3:**
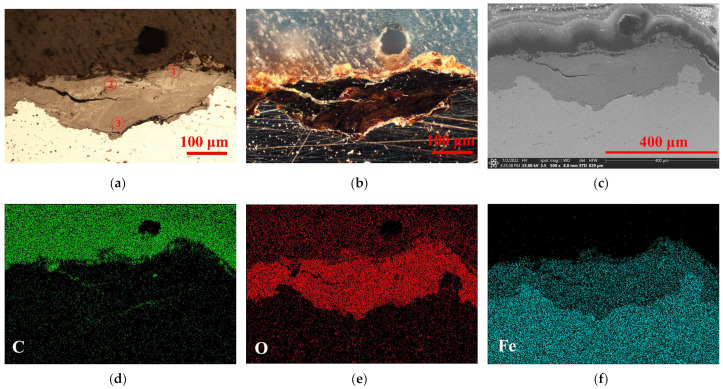
Original rust layer at area A. Optical microscopy in bright field (**a**); Optical microscopy in dark field (**b**); SEM picture (**c**); EDS of C, O, Fe elements (**d**–**f**).

**Figure 4:**
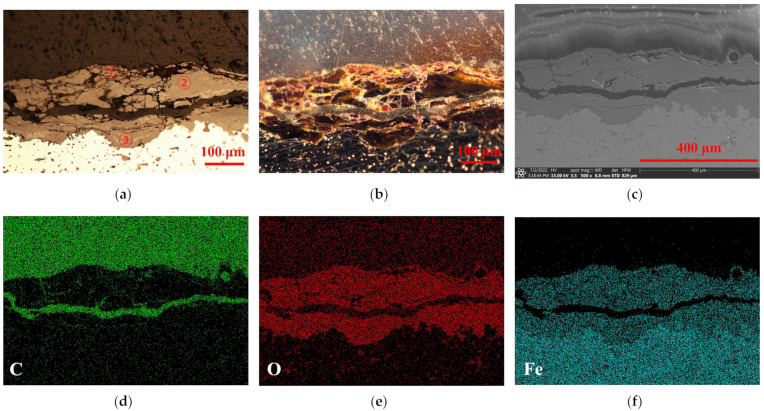
Original rust layer at area B. Optical microscopy in bright field (**a**); Optical microscopy in dark field (**b**); SEM picture (**c**); EDS of C, O, Fe elements (**d**–**f**).

Figure 3 and Figure 4 show the typical rust states and dominant elements’ distribution of this archaeological screw at area A and area B, respectively. The upper part was the resin layer, which had a transparent plastic texture under dark field (b), and was naturally rich in the C element of resin matrix. This could be convenient to determine the cross-sectional boundary of the surface rust. The central part was the key area of the rust layer. Under the bright field (a), the rust appeared to have delamination that was filled with O elements contained in various oxides or oxyhydroxides. The transition boundary between the metal core and the rust in the SEM image (c) was marked, and the Fe element filled the entire metal core. However, it could be seen that the cracking of these two rust layers of the archaeological screw was significantly different. One was overall intact with a crevice extending inward; the other one was full of tiny cracks and a large break grew horizontally, giving the outer rust an alarming tendency to fall off. Meanwhile, the barrier ability of area B to the external corrosive medium could be weaker than that of area A, making a worse corrosion state of it, which will be analyzed in the following experiments.

**Figure 5:**
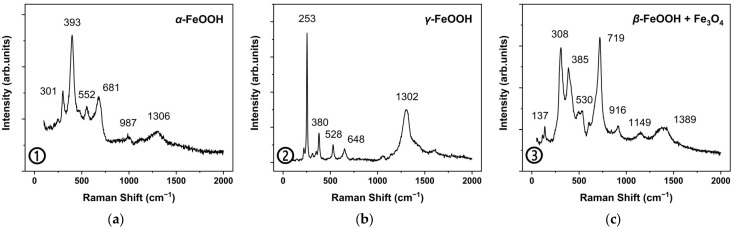
Micro-Raman diagrams of original rust area A. Testing points 1–3 (**a**–**c**) in Figure 3a.

The oxides and hydroxides of rust were identified by micro-Raman. Each rust layer was tested at three points from shallow to deep for basically determining the main components in the rust. In Figure 5, the point in the surface of area A showed a strong peak at 393 cm^−1^ with less intense peaks at 301, 552, 681 and 1306 cm^−1^ that were attributed to the existence of goethite (α-FeOOH) [38]. The lepidocrocite phase (γ-FeOOH) was linked to the strongest peak at 253 cm^−1^ and a second peak at 380 cm^−1^ that was formed in the light-colored area of the whole rust [39]. Akaganeite (β-FeOOH) was often observed at the dense oxides layer closed to the metal core. Due to its hygroscopicity, it was easy to retain acidic substances and salts in atmospheric rainwater, which was prone to transform into other oxides, especially the magnetite (Fe_3_O_4_) [3]. In area A, akaganeite had the bands at 137, 308 and 385 cm^−1^, while magnetite was assigned at 530, 719, 1149 and 1389 cm^−1^ near the steel core.

**Figure 6:**
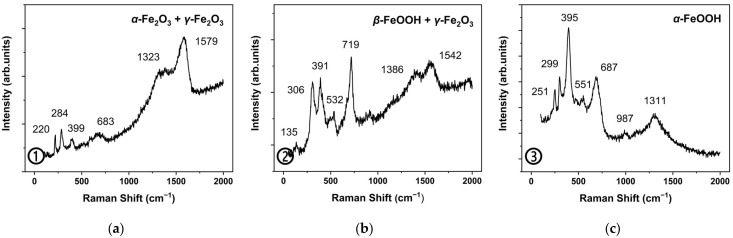
Micro-Raman diagrams of original rust area B. Testing points 1–3 (**a**–**c**) in Figure 4a.

In Figure 6, hematite (α-Fe_2_O_3_) and maghemite (γ-Fe_2_O_3_) were the dominant components in the outermost rust of area B, with characteristic peaks at 220, 284 and 399 cm^−1^ to α-Fe_2_O_3_ and the relatively weak peak at 683 and 1579 cm^−1^ to γ-Fe_2_O_3_. Akaganeite (β-FeOOH) and maghemite (γ-Fe_2_O_3_) both coexisted in the middle transition zone. Goethite (α-FeOOH) was identified in the light-white area in this point.

Therefore, goethite, lepidocrocite and akaganeite were the dominant phases in the whole rust layer. They could continue to maintain a process of transformation into oxides. With the internal stress in the transformation and the occurrence of electrochemical corrosion, the exfoliation of the corrosion products would be accelerated. These could make the rust have many cracks, so that the inner iron core still had channels in contact with the external environment, which was hard to be stable. If the above-mentioned oxyhydroxides could be stabilized and converted into inert compounds, these worrying phenomena would be gradually controlled, and the original information and research value of metal cultural heritage would be preserved to a greater behavior.

### 2.3. Rust Conversion

**Figure 7:**
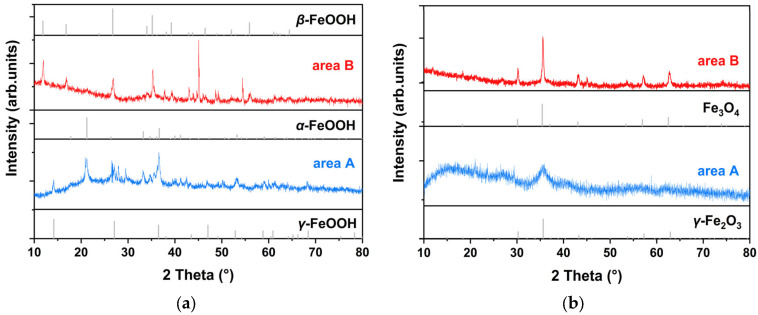
XRD of original rust (**a**) and the modified by proanthocyanidins (**b**).

The composition of original rust powder and the modification by proanthocyanidins (PC) were all analyzed by XRD in Figure 7. The data were compared respectively by Jade software and PDF-card references (α-FeOOH: #00-019-0713; β-FeOOH: #00-042-1315; γ-FeOOH: #00-008-0098; γ-Fe_2_O_3_: #00-039-1346; Fe_3_O_4_: #00-039-0629; Fe: #00-006-0696). For test area A, the fine correspondence between the peaks of the original rust and the standard spectrum of α-FeOOH and γ-FeOOH could be regarded as the main phases of rust. Meanwhile, for area B, a significant signal of β-FeOOH appeared, proving that there was a relatively obvious difference in the proportion of rust components between the two detection areas on the same artefacts, which also reflected the complex properties of real cultural heritage. There was a major peak of area B in Figure 7a, and it was attributed to iron or steel tiny particles compared with a PDF card obtained from scraping the archaeological steel. Ferric oxyhydroxides and oxides had various types, in which oxides could be regarded as the electrochemically stable phases [19]. Therefore, the composition in the rust powder preliminarily indicated that the rust phase of these archaeological irons had not yet reached stability and the subsequent stabilization treatment was necessarily required.

**Figure 8:**
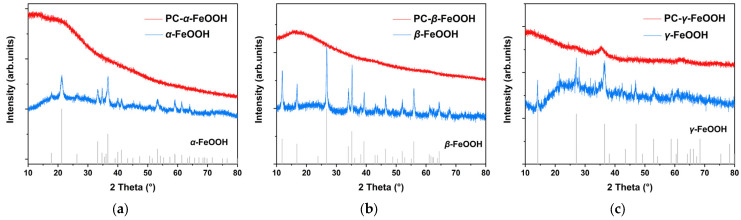
XRD of α-FeOOH (**a**), β-FeOOH (**b**), γ-FeOOH (**c**) modified by proanthocyanidins.

After proanthocyanidins modification, the XRD signals of archaeological rust were basically transformed into γ-Fe_2_O_3_ or Fe_3_O_4_, while the peaks of α-FeOOH, β-FeOOH and γ-FeOOH were extremely low and almost undetected in Figure 7b. This conversion showed a similar phenomenon in both area A and B, and area A presented a more pronounced state of amorphous products. To explain this result, Figure 8 compared the crystalline transformation results of three types of pure iron oxyhydroxides before and after PC treatment, respectively. FeOOH chemically reacted with PC, and the new product basically transformed the original crystalline state into an amorphous compound without marked diffraction peaks. Therefore, the active α, β and γ-FeOOH in the original rust could be consumed by the PC reaction, transforming it into amorphous solid substances and deposited in the rust. The relatively stable rust products Fe_2_O_3_ and Fe_3_O_4_ were not induced to show transformation so that they could still remain in the rust, which could be detected.

**Figure 9:**
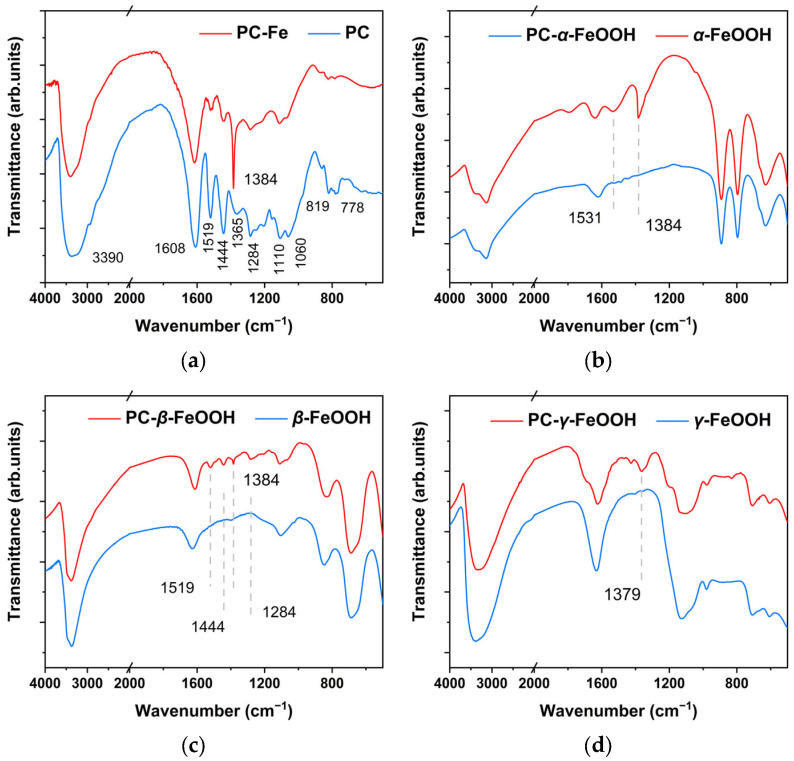
FTIR of original rust (**a**), α-FeOOH (**b**), β-FeOOH (**c**) and γ-FeOOH (**d**) modified by PC.

The infrared spectrum of proanthocyanidins is shown in Figure 9a. Many hydroxyl groups on this molecule could appear in the infrared peak of 3390 cm^−1^, which was attributed to the stretching vibration of −OH. The aromatic structure of this molecule was indicated by groups of peaks within wavenumbers in the range of 1000 to 2000 cm^−1^. The peak 1519 cm^−1^ was characteristic of aromatic ring C=C bond structure; 1444 cm^−1^ was attributed to −CH deformation; 1365 cm^−1^ could be assigned to the bending vibration of the C−O bond connected to the hydroxyl group on phenolic substances; 1284 cm^−1^ was the bending vibration of the C−O bond; 1110 cm^−1^ and 1060 cm^−1^ were the stretching vibration of the phenolic C−O−C bond. We found that 819 and 778 cm^−1^ were the bending vibration of =C−H bond on the out-of-plane benzene ring. The above functional groups were the basic parameters for identifying the presence of plant phenolic compounds [40]. The phenolic hydroxyl groups were prone to coordinate with metal cations to form a stable deposited structure [41]. The interaction between Fe^3+^ and the phenolic hydroxyl group had an effect on the O−C bond stretching vibration, in which the band shifted from 1365 cm^−1^ in the spectra of the PC to about 1384 cm^−1^ in the pure PC–Fe in Figure 9a. This phenomenon could be explained by the chelation and redox reaction of Fe^3+^ with phenolic hydroxyl.

In Figure 9b–d, infrared images show the conversion of three common iron oxyhydroxides under the proanthocyanidins modification. At the absorption position of 1384 cm^−1^, the reliable evidence for effective binding to PC molecules appeared, and the aromatic bonds of PC also showed a loading signal on the original FeOOH structure.

Therefore, with a combined analysis of XRD and FTIR, it could basically be considered that the oxyhydroxides on the archaeological steel chemically reacted with PC, and the active α, β, γ-FeOOH could be consumed and converted into an amorphous state as PC-FeOOH and deposited in the original rust, while the original relatively stable iron oxides were not induced to phase transformation and still remained.

**Figure 10:**
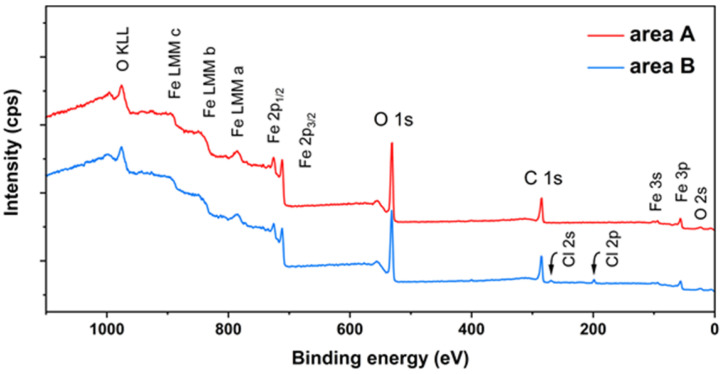
XPS survey of archaeological steel with proanthocyanidins.

Figure 10 shows the XPS survey of Fe 2p_3/2_, Fe 2p_1/2_, O 1s, and C 1s of the modified rust with PC. The trends of the two lines were almost the same, proving that the converted product tended to be uniform. For the peak of Cl element detected in area B, it could be divided into Cl 2s and Cl 2p. Combined with the results of XRD on the original rust at area B, it could be attributed to the structure of akaganeite, which had not yet fully reacted.

**Figure 11:**
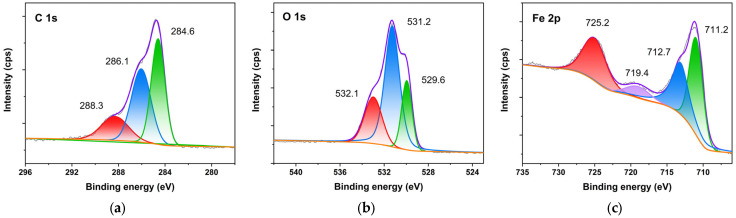
XPS spectra of rust in area A with proanthocyanidins. C 1s (**a**); O 1s (**b**); and Fe 2p (**c**).

For the area A in Figure 11, the detail of the C 1s spectrum was fitted into three peaks with binding energies at 284.6 eV, 286.1 eV and 288.3 eV, respectively. The peak at 284.6 eV was the signal of C−C, C=C and C−H. The peak at 286.1 eV could be related to the C−O−C on the organic materials, which should be the six-membered ring of PC. The appearance of the C−O complex with high binding energy at 288.3 eV was attributed to the presence of ether groups in PC-FeOOH [26]. The spectra of O 1s provided additional support for determination of the composition of converted rust. The O 1s spectrum was fitted into three peaks. The peak at 529.6 eV could be attributed to iron oxides, such as FeO, Fe_2_O_3_ and Fe_3_O_4_. This was consistent with the XRD results, again proving that PC could promote the consumption and conversion of the more active FeOOH, while keeping the oxides that remained in the original rust. The peak at 531.2 eV belonged to Fe−O, which was corresponding to the chelating structure formed by Fe^3+^ and the adjacent hydroxyphenyl groups on PC. The feature peak of 532.1 eV was assigned to the C−O−H bond in the organic PC structure [31]. Four peaks were fitted into the Fe 2p spectrum. The peak at 711.2 eV was FeO (Fe 2p_1/2_) and FeOOH complex. Fe_2_O_3_ was identified by the peak at 712.7 eV in Fe 2p_1/2_ and the peak at 725.2 eV in Fe 2p_3/2_. The peak at 719.4 eV was attributed to the adjunct of Fe^3+^ [42].

**Figure 12:**
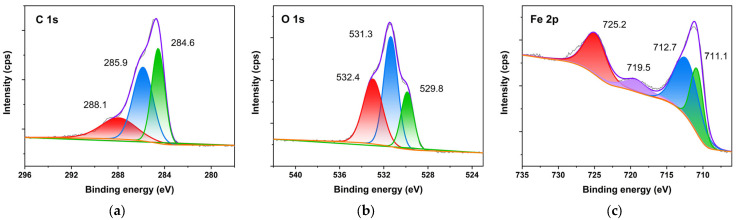
XPS spectra of rust in area B with proanthocyanidins. C 1s (**a**); O 1s (**b**); and Fe 2p (**c**).

For area B in Figure 12, the position of the main peak and the points of the sub-peaks were consistent with those of area A. Based on the analysis of FTIR and XRD spectra above, it was clear that PC have participated into the conversion of FeOOH without an obvious effect on iron oxides, which proved that the components of different original rust tend to be homogenized after being converted by PC.

### 2.4. Surface Morphology

**Figure 13:**
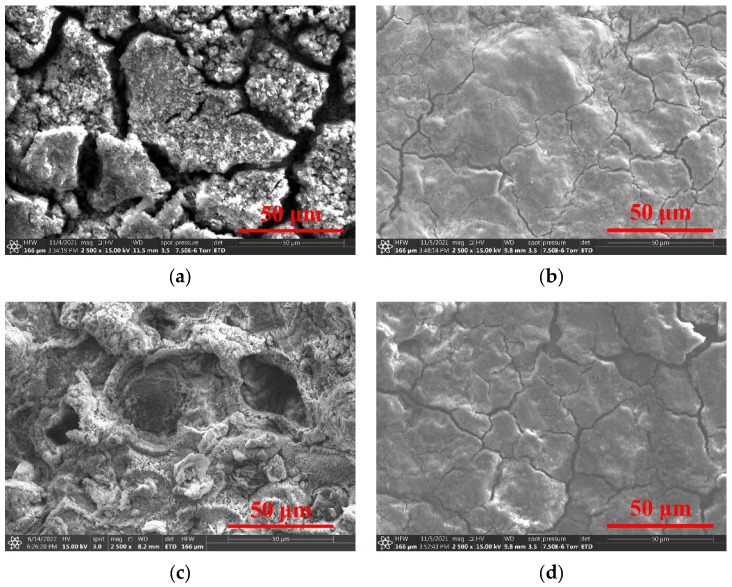
SEM of original surface in area A (**a**) and B (**c**); converted with PC in area A (**b**) and B (**d**).

The surface morphology of the archaeological rust layer was extremely complex and diverse. The results of the original area A and B showed obvious differences, as shown in Figure 13. The rust products in area A were not uniform in the particle sizes and formed irregular agglomeration. The rust layer was loose and rough with many wide cracks, and it was difficult to hinder the intrusion of external corrosion. The rust in area B was uneven and various rusts grew differently. The deep pits and holes had a tendency of rust peeling off.

Proanthocyanidins treatment could successfully convert the active rust into modified products and remain on the surface of archaeological steel. It gradually transformed the rust of different sizes, uneven distribution and various shapes into a flat laminated structure. The new layers were both compact and the cracks were narrowed in area A and B. Therefore, surface of the iron objects could be covered more uniformly, which was beneficial to delay the further corrosion of the internal iron core from the corrosive factors.

### 2.5. Electrochemical Analysis

**Figure 14:**
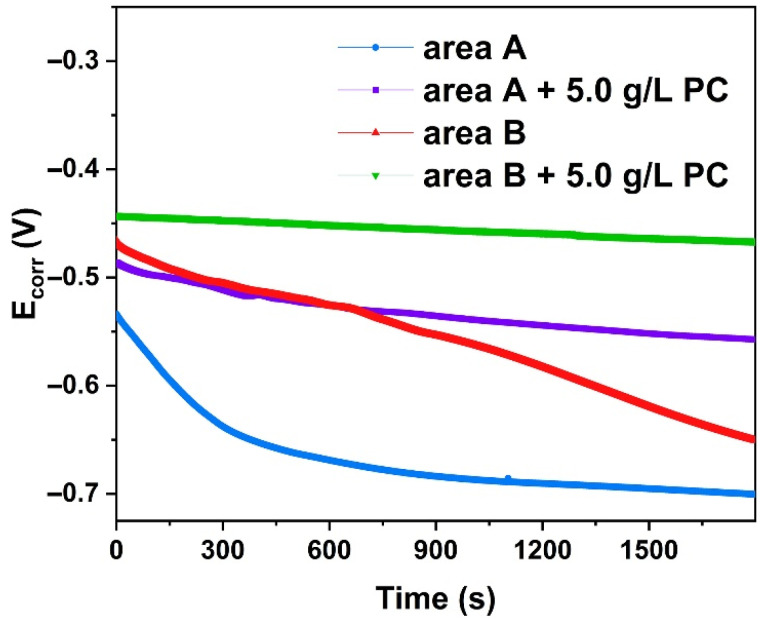
Corrosion potential of archaeological steel before and after conversion with PC.

Figure 14 is a comparison of the corrosion potential of archaeological steel before and after PC treatment. The original corrosion potential of area A could rapidly drop from −0.533 V to −0.701 V in 30 min without PC treatment, while the sample modified by PC would decrease from −0.486 V to −0.557 V. The drop was 42.26% of the original state. The corrosion potential of area B changed from −0.466 V to −0.651 V, and the addition of PC could slowly reduce the value from −0.442 V to −0.466 V, which was about 12.97% of the original state. This could prove that the converted rust layer of archaeological steel tended to be more stable in the corrosive medium and increased the corrosion potential more effectively than the original state.

**Figure 15:**
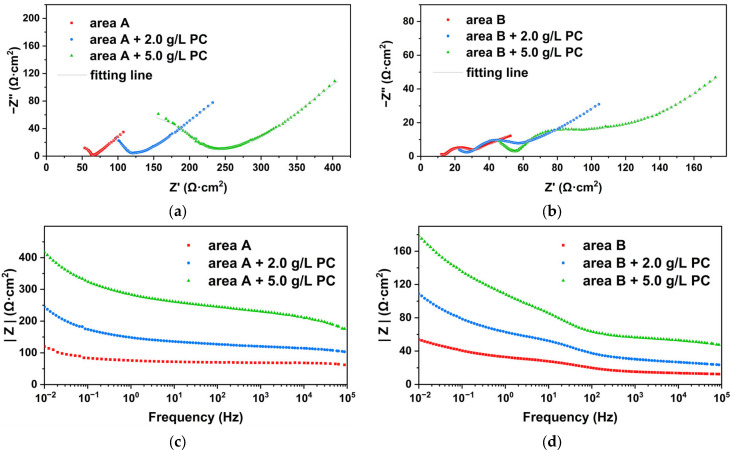

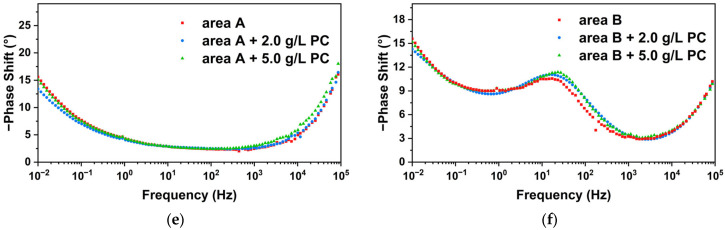
EIS plots of original and proanthocyanidins-treated archaeological steel in area A and B. Nyquist plots (**a**,**b**); |Z| vs. frequency in Bode plots (**c**,**d**); θ vs. frequency in Bode plots (**e**,**f**).

Due to long-term natural corrosion, archaeological steel has always been covered with thick rust deposited on the metal interface, thereby forming a barrier layer to ion diffusion. The external electrolyte and internal metal ions could basically have a slow diffusion through macroscopic pores and cracks, so that there was a long tail in the low frequency region representing typical Warburg impedance [43]. For both rusted areas, the Nyquist plots were composed of a depressed capacitive semi-arc that was assigned to the resistance of rust and electrolyte in the high frequency. At medium frequency, there were slight differences here. There was a semicircular transition region representing the charge transfer resistance of area B in Figure 15b, while the high and low frequency regions were almost directly transitioned in area A in Figure 15a. This meant that the corrosion state of area A was almost completely controlled by diffusion and area B was controlled by a mixture of charge transfer and ion diffusion.

**Figure 16:**
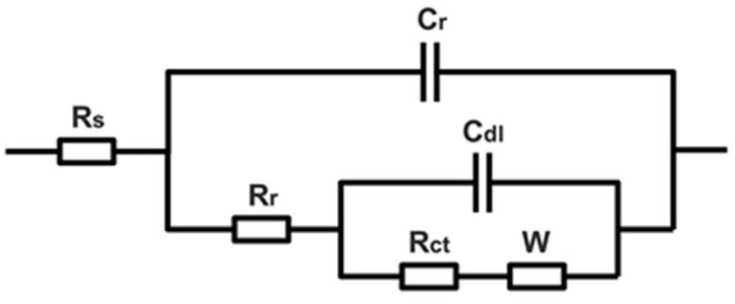
Equivalent circuits of EIS curves of archaeological steel.

Experimental data in Table 1 were fitted by the electrical equivalent circuits shown in Figure 16. R_s_ represented the electrolyte resistance, C_r_ the rust capacitance, R_r_ the rust resistance, C_dl_ the double-layer capacitance, R_ct_ the charge transfer resistance and W the Warburg resistance or barrier diffusion impedance. Due to the surface roughness and inhomogeneity, a constant phase angle element (CPE) was used to describe the C_dl_ and C_r_ in the fitting circuits [44]. After the archaeological steel was soaked with PC, the radius of the semi-arc in the high-frequency region become significantly larger, which indicated that PC effectively increased the resistance value of the rust layer. We found that 2.0 g/L of PC could increase the R_r_ from 34.39 Ω·cm^2^ to 63.69 Ω·cm^2^, while 5.0 g/L of PC could increase the R_r_ to 207.76 Ω·cm^2^ by 6 times in area A. The same occurred in area B: R_r_ was from 15.74 Ω·cm^2^ to 56.16 Ω·cm^2^ by 3.5 times under the modified with 5.0 g/L of PC. It was proved that the modified rust by PC was more inert and less conductive in corrosive media. The capacitance value CPE_r_ and n_r_ of the rust decreased with the increase of the concentration of PC, which could indicate that the PC reacted with the original rust to generate new compounds that could effectively hinder the penetration of corrosive media. Meanwhile, a CPE with *n* value about 0.5 could be used to produce an infinite-length Warburg element. The infinite-length Warburg element occurred when the charge carrier diffused through a material, especially in the medium and low frequencies. If the rust layer was thin, low frequencies would penetrate the entire thickness, creating the finite-length Warburg element. If the rust layer was thick enough, the low frequencies applied did not fully penetrate the layer, and it could be used as an infinite-length Warburg element [45]. The n_r_ gradually approached 0.5 with the support of PC, which meant that the new products had filled the loose and porous structure of the original rust layer, resulting in a dense, even and uniform surface. Then, the R_ct_ in the medium frequency was also increased and CPE_dl_ dropped gradually due to the PC addition. We found that 5.0 g/L of PC could increase the R_ct_ from 15.32 Ω·cm^2^ to 98.34 Ω·cm^2^ by 6.4 times in area A, while to area B, R_ct_ was from 12.38 Ω·cm^2^ to 31.92 Ω·cm^2^ by 2.6 times. This was because the PC pretreatment could improve the protection of internal metal and increased the charge transfer resistance of rust, indicating that PC could modify rust to be denser than the original. The Warburg impedance in the low frequency region was also significantly improved, which meant that the modified rust layer had the ability to effectively block the charge and ion transfer between the rusted surface and the corrosive solution, for improving the protection of the internal metal.

## 3. Materials and Methods

### 3.1. Archaeological Objects

**Figure 17:**
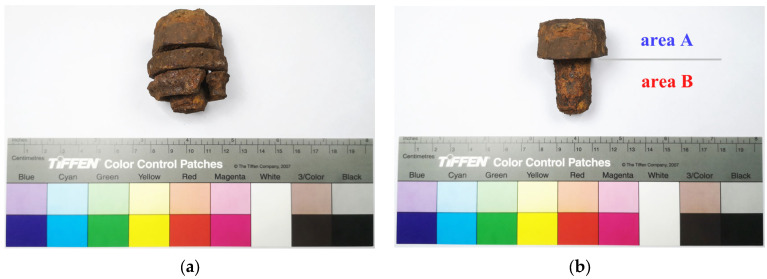
Archaeological steel screw from Lingzhao Xuan of the Qing Dynasty in the Forbidden City. Original state (**a**); After separation (**b**).

To fully prove the effect of proanthocyanidins on the rust transformation and corrosion inhibition of real iron cultural heritage, a sample was taken from the steel architecture, which could be called Lingzhao Xuan (Wondrous Pond Pavilion) of the Qing Dynasty in the Forbidden City. Many original components were missing due to severe corrosion, so the dropped screw was extracted from the ground floor of the architecture as a sample for this experiment. The initial state of the screw was stacked with two C-rings to form a whole due to rust and sand (in Figure 17), and they were successfully peeled and separated in the pre-conservation in Figure 17b. During initial cleaning with deionized water to remove the silt and mud, the rust layers were gradually exposed. In the test, the upper hexagonal prism structure of the screw was used as the experimental area A, while the lower cylindrical structure was used as the experimental area B to realize the full utilization of the sample. The surface appearance and the approximate dimension are all shown in Figure 17.

### 3.2. Samples Preparation

#### 3.2.1. Rust Powder

##### Synthesis of PC-FeOOH

The α-FeOOH was prepared by 5.0 wt.% of FeSO_4_ and 1.0 wt.% of NaOH with de-ionized water. The solution was bubbled with oxygen for 12 h under 60 °C and pH was to 13. The precipitates were washed with deionized water until the pH was neutral before being dried at 85 °C.

The β-FeOOH was prepared by 5.0 wt.% of FeCl_3_ with deionized water kept for 12 h under 60 °C. 0.5 wt.% of EDTA and 5 mL of ammonia was added to form dark-red precipitates. The mixture was washed with deionized water and dried at 85 °C.

The γ-FeOOH was prepared by 5.0 wt.% of FeCl_2_⋅4H_2_O, 5.0 wt.% of urotropine and 2.0 wt.% of NaNO_2_ in deionized water. The mixture was kept constant stirring under 60 °C for 12 h. The precipitates were washed and dried at 85 °C.

For testing the interaction between PC and FeOOH, 1.0 wt.% of each α, β, γ-FeOOH was added into 5.0 g/L of PC solution in plastic tubes, respectively. The tubes were sealed and shaken to ensure complete reaction for 72 h. The products were washed with deionized water and dried at 85 °C for forming PC-FeOOH powder. Proanthocyanidins (PC) were obtained from MREDA Technology Co., Beijing, China.

##### Modification of Original Rust

The original rust was scraped from the steel object with a razor at the areas A and B, respectively. They were immersed in 5.0 g/L of PC solution with distilled water for 72 h. The products were washed and dried at 85 ℃ for forming modified rust.

All the powder samples were milled in an agate mortar for a uniform and fine size.

#### 3.2.2. Metallographic Samples

The samples were cut from the edge at areas A and B of the steel screw. After being dried at room temperature, they were embedded in epoxy resin for mechanically grinding by SiC paper (grade 80~2000) under absolute ethanol. Both samples were polished with diamond paste to obtain a smooth and shiny metal surface. The surface was etched with 4.0% (volume ratio) HNO_3_ alcohol reagent to facilitate subsequent observation.

#### 3.2.3. Working Electrodes

The objects to be tested were cut into small pieces to ensure that the detection area was 10 mm × 10 mm. To keep the original state of the archaeological rust layer and the iron core better and the data repeatability, we tried to cut continuously along one side. They were carefully embedded in epoxy to mask the cut edges and protect the electrochemical impedance test from noise interference. The original state and soaking treatment of the samples were all dried before being infused with resin.

### 3.3. Morphology Observation

An optical stereomicroscope (Eclipse LV100ND, Nikon, Tokyo, Japan) equipped with a digital camera (DS-Ri2, Nikon, Tokyo, Japan) was used to observe the metallographic structure of the steel core and the appearance of the rust layers.

A scanning electron microscope (Quattro ESEM, Thermo Fisher Scientific, Waltham, MA, USA) with an energy dispersive spectroscopy (Quattro ESEM EDS, Thermo Fisher Scientific, Waltham, MA, USA) was used to show the morphology of samples and elements composition of metal core in details under an accelerating voltage of 15 kV. For alloy composition of this archaeological steel screw, EDS was chosen to use mapping mode to obtain 3 points data and perform average calculation.

### 3.4. Determination of Rust Conversion

X-ray diffraction was recorded by a diffractometer (X’pert-3 Power, PANanalytical, Almelo, The Netherlands) with a Cu anode, in order to determine the crystalline structure of the rust powder. The generator voltage used was 40 kV, while the tube current was 40 mA. Angular scanning was performed from 5° to 80°, with a scan step size of 0.013°.

FTIR transmittance analysis (Tensor 27, Bruker, Karlsruhe, Germany) was prepared by KBr pellets to define chemical structures of rust in the range from 400 to 4000 cm^−1^ at 4 cm^−1^ resolution. A powder sample of 0.2 mg was mixed with 200 mg of dry KBr (>99% FTIR grade, Sigma-Aldrich, Burlington, MA, USA) to be pressed into pellets.

Micro-Raman spectroscopy was performed on the cross-sectional sample using a micro-Raman spectroscope (Thermo Scientific DXRxi, Thermo Fisher Scientific, Waltham, MA, USA) that was equipped with a microscope (OLYMPUS BX51, Olympus Corporation, Tokyo, Japan) to define different rust components. Raman excitation was provided by a frequency-doubled Nd: YAG laser operating at 532 nm, with a power of about 0.2 mW and with a probe diameter of about 1.0 mm. All spectra were calibrated using 519.8 cm^−1^ line of silicon wafer.

The elemental composition and valence state were investigated by X-ray photoelectron spectrometer (AXIS Supra, Kratos Analytical Ltd., Manchester, UK). Aluminum was used as X-ray photon source with a power of 150 W. The pass energy in full spectrum was set at 160 eV and energy step was 1.0 eV. The pass energy in C, O, Fe elemental spectra were set at 40 eV and energy step was 0.1 eV.

### 3.5. Electrochemical Impedance

Electrochemical impedance spectroscopy of rusted and naked archaeological iron objects was conducted using an electrochemical workstation (CS-350, CorrTestTM, Wuhan, China). Measurements were carried out in a three-electrode cell with 3.5 wt.% NaCl electrolyte to simulate accelerated corrosion. The three-electrode cell included a saturated calomel reference electrode; a platinum auxiliary electrode with an exposure surface of 10 mm × 10 mm; the steel objects with an exposure surface of 10 mm × 10 mm functioned as working electrodes. Before testing, the electrodes were kept in the solution for 30 min to stabilize the free corrosion potential. An open-circuit potential was applied with frequencies ranging from 100 kHz to 10 mHz, and a sinusoidal perturbation signal with a 10 mV amplitude was used. The obtained data were interpreted based on an equivalent circuit using View (Zview2, Scribner Associates Inc., Southern Pines, NC, USA) to obtain the fitting parameters.

## 4. Conclusions

To explore the behavior of eco-friendly proanthocyanidins on the rust conversion and corrosion inhibition of real archaeological iron, the sample was a steel screw from the Lingzhao Xuan (Wondrous Pond Pavilion) of the Qing Dynasty in the Forbidden City. In the test, the upper hexagonal prism of the screw was used as the experimental area A, while the lower cylindrical structure was used as the area B to fully utilize the sample.

The archaeological screw was hypoeutectoid steel with a mass ratio of main elements of 0.24% C, 0.06% Si, 0.15% P, 0.16% S and 92.95% Fe. The main phases of rust were the α-FeOOH and γ-FeOOH for test area A, while β-FeOOH was regarded as the dominant component analyzed by XRD and Raman spectroscopy. After proanthocyanidins modification, the XRD signals of archaeological rust were basically transformed into γ-Fe_2_O_3_ or Fe_3_O_4_, while the peaks of α, β, γ-FeOOH were extremely low and almost undetected.

To explain this phenomenon, three types of pure iron oxyhydroxides as samples could prove that proanthocyanidins had the ability to chemically react with FeOOH, and the active α, β, γ-FeOOH could be consumed and converted into an amorphous state as PC-FeOOH deposited in the original rust without marked diffraction peaks. The relatively stable iron oxides were not induced to show transformation and still remained in the rust that could be detected. At the absorption position of 1384 cm^−1^, the reliable evidence for effective binding to PC molecules appeared, and the aromatic bonds of PC also showed a loading signal on the original FeOOH structure analyzed by XRD, XPS and FTIR.

The improved corrosion inhibition of the modified rust layer was detected by SEM, EIS and corrosion potential. The corrosion state of the area A was almost completely controlled by diffusion, and the area B was controlled by a mixture of charge transfer and ion diffusion. The modification of PC did not change the mode of corrosion control of the archaeological steel. The converted rust filled the loose and porous structure of the original rust layer, resulting in a dense, even and uniform surface, which could be more stable in the corrosive medium and increased the corrosion potential more effectively. Both the rust layer resistance and the charge transfer resistance of the archaeological samples was improved by about 3 times with 5.0 g/L of PC, which could reasonably stabilize the archaeological rust and hinder external corrosive penetration into the core.

The original and complex state of the metal heritage samples complicated the selection of actual conservation reagents. Proanthocyanidins could promote the stabilization and conversion of the rust layer, and effectively inhibit the corrosion of the steel core. It has a good application prospect as an eco-friendly protection material for steel cultural heritage.

## Figures and Tables

**Table 1:** Fitting parameters of EIS curves of archaeological steel.

Samples		R_s_ (Ω·cm^2^)	R_r_ (Ω·cm^2^)	R_ct_ (Ω·cm^2^)	R_w_ (Ω·cm^2^)	CPE_r_ (s^n^·Ω^−1^·cm^−2^)	CPE_dl_ (s^n^·Ω^−1^·cm^−2^)	n_r_	n_dl_	χ^2^
area A	blank	11.69	34.39	15.32	27.23	1.73 × 10^−7^	16.91 × 10^−4^	0.84	0.42	2.03 × 10^−4^
	2.0 g/L	12.52	63.69	56.23	83.34	1.64 × 10^−7^	4.73 × 10^−4^	0.71	0.44	2.45 × 10^−4^
	5.0 g/L	11.05	207.76	98.34	275.65	0.31 × 10^−7^	4.40 × 10^−4^	0.66	0.39	2.61 × 10^−4^
area B	blank	13.31	15.74	12.38	84.42	5.30 × 10^−4^	7.98 × 10^−4^	0.75	0.37	4.51 × 10^−4^
	2.0 g/L	10.75	31.71	23.44	148.32	1.91 × 10^−4^	6.27 × 10^−4^	0.73	0.41	1.62 × 10^−4^
	5.0 g/L	11.22	56.16	31.92	182.36	0.04 × 10^−4^	6.10 × 10^−4^	0.71	0.54	1.14 × 10^−4^

## Data Availability

The data presented in this study are available in the article.

## References

[1] Jasniok T., Jasniok M., Skorkowski A. (2021). Diagnostics of Large Non-Conductive Anti-Corrosion Coatings on Steel Structures by Means of Electrochemical Impedance Spectroscopy. Materials.

[2] Vasconez-Maza M.D., Martinez-Pagan P., Aktarakci H., Garcia-Nieto M.C., Martinez-Segura M.A. (2020). Enhancing Electrical Contact with a Commercial Polymer for Electrical Resistivity Tomography on Archaeological Sites: A Case Study. Materials.

[3] Calero J., Alcantara J., Chico B., Diaz I., Simancas J., de la Fuente D., Morcillo M. (2017). Wet/dry accelerated laboratory test to simulate the formation of multilayered rust on carbon steel in marine atmospheres. Corros. Eng. Sci. Technol..

[4] Qian B., Hou B., Zheng M. (2013). The inhibition effect of tannic acid on mild steel corrosion in seawater wet/dry cyclic conditions. Corros. Sci..

[5] Aourabi S., Driouch M., Sfaira M., Mahjoubi F., Hammouti B., Verma C., Ebenso E.E., Guo L. (2021). Phenolic fraction of Ammi visnaga extract as environmentally friendly antioxidant and corrosion inhibitor for mild steel in acidic medium. J. Mol. Liq..

[6] Luo Q., Zhang J.-R., Li H.-B., Wu D.-T., Geng F., Corke H., Wei X.-L., Gan R.-Y. (2020). Green Extraction of Antioxidant Polyphenols from Green Tea (*Camellia sinensis*). Antioxidants.

[7] Domenech A., Lastras M., Rodriguez F., Osete L. (2013). Mapping of corrosion products of highly altered archeological iron using voltammetry of microparticles. Microchem. J..

[8] Reitzer F., Allais M., Ball V., Meyer F. (2018). Polyphenols at interfaces. Adv. Colloid Interface Sci..

[9] Chun Q., Zhang J.W., Zhao P., Han Y.D., Meng Z. (2018). Research on damage to and structural performance of Lingzhao Xuan in the Forbidden City. Sci. Consev. Archaeol..

[10] Zhou Q., Yan W.M., Ji J.B. (2016). Dynamic characteristic and seismic responses of steel structure of Ling-zhao Veranda in the Palace Museum. J. Shandong Univ. Eng. Sci..

[11] Caponetti E., Francesco A., Martino D.C., Saladino M.L., Ridolfi S., Chirco G., Berrettoni M., Conti P., Bruno N., Tusa S. (2017). First discovery of orichalcum ingots from the remains of a 6th century bc shipwreck near Gela (Sicily) seabed. Mediterr. Archaeol. Archaeom..

[12] Quaranta M., Catelli E., Prati S., Sciutto G., Mazzeo R. (2014). Chinese archaeological artefacts: Microstructure and corrosion behaviour of high-leaded bronzes. J. Cult. Herit..

[13] Ingo G.M., De Caro T., Riccucci C., Angelini E., Grassini S., Balbi S., Bernardini P., Salvi D., Bousselmi L., Cilingiroglu A. (2006). Large scale investigation of chemical composition, structure and corrosion mechanism of bronze archeological artefacts from Mediterranean basin. Appl. Phys. A.

[14] Grevey A.L., Vignal V., Krawiec H., Ozga P., Peche-Quilichini K., Rivalan A., Maziere F. (2020). Microstructure and long-term corrosion of archaeological iron alloy artefacts. Herit. Sci..

[15] Bernabale M., Nigro L., Vaccaro C., Nicoli M., Montanari D., Bigini P., De Vito C. (2022). Micro-Raman spectroscopy and complementary techniques for the study of iron weapons from Motya and Lilybaeum (Sicily, Italy): Corrosion patterns in lagoon-like and calcarenitic hypogea environments. J. Raman Spectrosc..

[16] Morcillo M., Wolthuis R., Alcantara J., Chico B., Diaz I., de la Fuente D. (2016). Scanning Electron Microscopy/Micro-Raman: A Very Useful Technique for Characterizing the Morphologies of Rust Phases Formed on Carbon Steel in Atmospheric Exposures. Corrosion.

[17] Letardi P., Salvadori B., Galeotti M., Cagnini A., Porcinai S., Santagostino Barbone A., Sansonetti A. (2016). An in situ multi-analytical approach in the restoration of bronze artefacts. Microchem. J..

[18] Hoerle S., Mazaudier F., Dillmann P., Santarini G. (2004). Advances in understanding atmospheric corrosion of iron. II. Mechanistic modelling of wet-dry cycles. Corros. Sci..

[19] Nishimura T., Katayama H., Noda K., Kodama T. (2000). Electrochemical behavior of rust formed on carbon steel in a wet/dry environment containing chloride ions. Corrosion.

[20] Estalayo E., Aramendia J., Mates Luque J.M., Manuel Madariaga J. (2019). Chemical study of degradation processes in ancient metallic materials rescued from underwater medium. J. Raman Spectrosc..

[21] Hu P., Jia M.H., Li M.H., Sun J., Cui Y., Hu D.B., Hu G. (2022). Corrosion Behavior of Ancient White Cast Iron Artifacts from Marine Excavations at Atmospheric Condition. Metals.

[22] Lair V., Antony H., Legrand L., Chausse A. (2006). Electrochemical reduction of ferric corrosion products and evaluation of galvanic coupling with iron. Corros. Sci..

[23] Artesani A., Di Turo F., Zucchelli M., Traviglia A. (2020). Recent Advances in Protective Coatings for Cultural Heritage-An Overview. Coatings.

[24] Flores Merino S., Jose Caprari J., Vasquez Torres L. (2017). Inhibitive action of tara tannin in rust converter formulation. Anti-Corros. Methods Mater..

[25] Alsabagh A.M., Migahed M.A., Abdelraouf M., Khamis E.A. (2015). Utilization of Green Tea as Environmentally Friendly Corrosion Inhibitor for Carbon Steel in acidic media. Inter. J. Electrochem. Sci..

[26] Wang D.Y., Nie B.L., Li H.J., Zhang W.W., Wu Y.C. (2021). Anticorrosion performance of grape seed proanthocyanidins extract and Tween-80 for mild steel in hydrochloric acid medium. J. Mol. Liq..

[27] Grigsby W.J. (2017). Simulating the protective role of bark proanthocyanidins in surface coatings: Unexpected beneficial photo-stabilisation of exposed timber surfaces. Prog. Org. Coat..

[28] Chen J., Chen Y.X., Zheng Y.F., Zhao J.W., Yu H.L., Zhu J.J. (2022). The Relationship between Procyanidin Structure and Their Protective Effect in a Parkinson’s Disease Model. Molecules.

[29] Habib H.M., El-Fakharany E.M., Kheadr E., Ibrahim W.H. (2022). Grape seed proanthocyanidin extract inhibits DNA and protein damage and labile iron, enzyme, and cancer cell activities. Sci. Rep..

[30] Wang D.Y., Li H.J., Chen X., Nie B.L., Wu Y.C. (2022). Fabricating of grape seed proanthocyanidins loaded Zein-NaCas composite nanoparticles to exert effective inhibition of Q235 steel corrosion in seawater. J. Mol. Liq..

[31] Huang L., Yang K.P., Zhao Q., Li H.J., Wang J.Y., Wu Y.C. (2022). Corrosion resistance and antibacterial activity of procyanidin B2 as a novel environment-friendly inhibitor for Q235 steel in 1 M HCl solution. Bioelectrochemistry.

[32] Guedes D., Martins G.R., Jaramillo L.Y.A., Bernardes Dias D.S., da Silva A.J.R., Lutterbach M.T.S., Reznik L.Y., Servulo E.F.C., Alviano C.S., Alviano D.S. (2021). Proanthocyanidins with Corrosion Inhibition Activity for AISI 1020 Carbon Steel under Neutral pH Conditions of Coconut (*Cocos nucifera* L.) Husk Fibers. ACS Omega.

[33] Hussin M.H., Kassim M.J. (2011). The corrosion inhibition and adsorption behavior of Uncaria gambir extract on mild steel in 1 M HCl. Mater. Chem. Phys..

[34] Abdallah M. (2002). Rhodanine azosulpha drugs as corrosion inhibitors for corrosion of 304 stainless steel in hydrochloric acid solution. Corros. Sci..

[35] Tang Z. (2019). A review of corrosion inhibitors for rust preventative fluids. Curr. Opin. Solid State Mater. Sci..

[36] Zhao X.D., Cheng Y.F., Fan W., Vladimir C., Volha V., Alla T. (2014). Inhibitive Performance of a Rust Converter on Corrosion of Mild Steel. J. Mater. Eng. Perform..

[37] Mohapatra J.N., Babu T.S., Dabbiru S.K., Balachandran G. (2021). Magnetic Hysteresis Loop as a Tool for the Evaluation of Mechanical Properties of Hypoeutectoid Pearlitic Steels with Spheroidization Heat Treatmemt. J. Nondestr. Eval..

[38] Saleh S.A. (2017). Corrosion Mechanism of Iron Objects in Marine Environment an Analytical Investigation Study by Raman Spectrometry. Eur. J. Sci. Theol..

[39] Rocca E., Faiz H., Dillmann P., Neff D., Mirambet F. (2019). Electrochemical behavior of thick rust layers on steel artefact: Mechanism of corrosion inhibition. Electrochim. Acta.

[40] Selvi I.K., Nagarajan S. (2018). Separation of catechins from green tea (*Camellia sinensis* L.) by microwave assisted acetylation, evaluation of antioxidant potential of individual components and spectroscopic analysis. LWT.

[41] Zhang R.N., Li L., Liu J.X. (2015). Synthesis and characterization of ferric tannate as a novel porous adsorptive-catalyst for nitrogen removal from wastewater. RSC Adv..

[42] Xu W.H., Han E.H., Wang Z.Y. (2019). Effect of tannic acid on corrosion behavior of carbon steel in NaCl solution. J. Mater. Sci. Technol..

[43] Zhang X., Yang S.W., Zhang W.H., Guo H., He X.L. (2014). Influence of outer rust layers on corrosion of carbon steel and weathering steel during wet-dry cycles. Corros. Sci..

[44] Han D., Jiang R.J., Cheng Y.F. (2013). Mechanism of electrochemical corrosion of carbon steel under deoxygenated water drop and sand deposit. Electrochim. Acta.

[45] Orazem M.E., Frateur I., Tribollet B., Vivier V., Marcelin S., Pebere N., Bunge A.L., White E.A., Riemer D.P., Musiani M. (2013). Dielectric Properties of Materials Showing Constant-Phase-Element (CPE) Impedance Response. J. Electrochem. Soc..

